# Surgical and Bronchoscopic Lung Volume Reduction in Chronic Obstructive Pulmonary Disease

**DOI:** 10.1155/2014/757016

**Published:** 2014-12-30

**Authors:** Manoj Meena, Ramakant Dixit, Mrityunjaya Singh, Jai Kumar Samaria, Surendra Kumar

**Affiliations:** ^1^Department of Respiratory Medicine, JLN Medical College, Ajmer, Rajasthan 305001, India; ^2^Department of Tuberculosis & Respiratory Diseases, IMS, BHU, Varanasi, Uttar Pradesh 221005, India; ^3^Department of Dermatology and Venereology, JLN Medical College, Ajmer, Rajasthan 305001, India

## Abstract

Chronic obstructive pulmonary disease (COPD) is the most extensively studied and researched disease in pulmonology and a cause of significant morbidity, mortality, and financial burden on patient's family and country's economy. Its management continues to be a challenge to both the physician and the patient's family. So far, it is preventable and treatable but not curable. Emphysema, a phenotype of COPD, is the most debilitating condition associated with progressive exercise intolerance and severe dyspnea. Despite decades of research, medical treatments available so far have helped improve quality of life and slowed down the decline in respiratory function but did not significantly improve the survival benefits. Though surgical lung volume reduction (LVR) procedures have shown some promise in context to functional gains and survival but, only in a carefully selected group of patients, bronchoscopic LVR procedures are yet to explore their full potential and limitations. This paper retrospectively studied the developments so far, medical and surgical, with special emphasis on the bronchoscopic procedures of lung volume reduction, and tried to comparatively analyze the risks and benefits of each one of them through various trials and studies done to date.

## 1. Chronic Obstructive Pulmonary Disease: Available Treatments and Interventions

Chronic obstructive pulmonary disease is now a widely recognized major health problem with an increasing trend throughout the globe. As per the future projections it is likely to become the third leading cause of death [1] by year 2030 and fifth leading cause of DALY (Disability Adjusted Life Years) lost worldwide by year 2020 [2]. A more recent study predicts it to be the fourth leading cause of death by 2030 [3].

Our understanding of the disease so far has led us to believe that COPD behaves like a condition with premature aging of the lungs and that it is more of a systemic inflammatory disease with lungs as its primary target. This is evident from the fact that the natural course of COPD is characterized by a persistent fall in pulmonary function (FEV_1_) two-to-three times faster than seen in normal aging nonsmoking population [4] resulting in disabling symptoms, reduced exercise capacity, poor health related quality of life (HRQoL), chronic respiratory failure, and premature death. Inflammation remains a cornerstone in development and pathogenesis of COPD and its comorbidities [5, 6] and studies have shown various specific and nonspecific inflammatory markers increased in lungs and systemic circulation in patients with COPD and particularly in smokers. Recent studies have added emphasis to oxidative stress as a significant contributing factor to the disease process which is increased severalfold particularly during acute exacerbations [7].

COPD is a potentially preventable and treatable disease. Tobacco (cigarette and other forms) smoking is by far the most implicated and discussed culprit; though not all patients develop COPD (only one out of three to four) but nearly 90% of COPD are smokers [8]. Among other risk factors are organic and inorganic chemical agents, dusts and fumes, indoor pollution from biomass fuel smoke, poor socioeconomic status, bronchial asthma, bronchial hypersensitivity, chronic bronchitis, early childhood infections, and pulmonary tuberculosis [9].

The current management of COPD is largely based on the understanding of its pathophysiology which is a cellular (neutrophil, macrophage, and cytotoxic CD8+ Tc1 lymphocyte) and chemically (cytokines, growth factors, and chemotactic factors) driven inflammatory response which leads to an oxidant-antioxidant and protease-antiprotease imbalance [5]. The resulting changes from structural remodeling, that is, chronic bronchitis (large airways), bronchiolitis (small airways), and pulmonary emphysema (lung parenchyma), lead to reduced airway resistance and decreased elastic recoil of lung which in turn leads to the characteristic expiratory airflow limitation. A direct result of this airflow limitation is air-trapping and hyperinflation further aggravated during exercise (dynamic hyperinflation). This dynamic component better explains the patient's symptoms such as dyspnea and exercise limitation [10] than the spirometric degree of airflow limitation itself [11]. The airflow limitation is progressive and only partly reversible and is further accelerated by repeated acute exacerbations (AE) [12, 13].

Medical management of COPD largely relies on smoking cessation, bronchodilator therapy, anti-inflammatory drugs, antibiotics, prophylactic vaccination, pulmonary rehabilitation and domiciliary oxygen therapy, mucolytics, antioxidants, and nutritive support among others. In order to rationalize the management, evidence based “Global Initiatives for Management of Obstructive Lung Diseases” (GOLD) guidelines have been formulated. It proposes management in a staged manner, stages I to IV based on severity of airflow limitation (as shown from postbronchodilator FEV1 expressed as a percentage of predicted value). Of all the therapies, smoking cessation is the only intervention shown to have a disease modifying effect that can slow down the decline in lung function and increase survival [14]. Nicotine substitution therapy and drugs such as* Bupropion* and* Varenicline* are useful for this. First line recommended bronchodilator for maintenance therapy is long acting muscarinic antagonists (LAMA);* Tiotropium* preferably combined with a long acting *β*_2_ receptor agonist (LABA) such as* Formoterol* or* Salmeterol*. Ultralong acting LABAs (effective up to 24 hr) like* Indacaterol* and* Arformoterol* are also available. Other ultralong acting LABAs like* Olondaterol* and* Vilanterol* are to be launched soon. Role of inhaled corticosteroids in COPD though common in practice is still highly debated because the type of inflammation in COPD in contrast to asthma is not particularly responsive to inhaled corticosteroids (ICSs) therapy. This is largely due to reduced activity of enzyme* histone deacetylase-2* (which is responsible for reversing the acetylation of activated inflammatory genes after getting recruited by activated glucocorticoid receptors) which can be attributed to smoking induced inflammation and oxidative stress [15, 16]. ICSs however still find place in GOLD recommendation owing to studies showing reduction in acute exacerbation rates and improvement in symptoms and lung functions [17]. Oral corticosteroids are beneficial as short course therapy in acute exacerbations only [18]. The associated comorbidities should essentially be treated as per their recommended guidelines and no alteration in either treatment is usually necessary when COPD and a comorbidity together exist.

Despite proper medical therapy, a large number of patients with advanced disease have been seen to deteriorate quite rapidly in their course or achieve minimal or no control. Management of such patients includes a more radical approach like lung transplantation and lung volume reduction surgeries, taking into account the facilities and expertise available and cost issues and patient's fitness for surgery. Bronchoscopic lung volume reduction (BLVR) procedures have lately made heads turn and offer to add a new dimension to treatment modalities in COPD. It may however be noted that LVR offers no role so far in treatment of nonemphysematous COPD and no studies/trials have been done for the same.

## 2. Role of Lung Volume Reduction (LVR)

The word “lung volume reduction” in the initial days of its conception hypothesized the reduction (by removal) of that portion of the lung which is supposed to be physiologically nonfunctional, that is, which does not participate in or renders ineffective the normal process of gaseous exchange between air in the alveoli and blood in the alveolar capillaries. This portion of lung might also physically compromise the function of normal lung tissue adjacent to it by compression.

Surgical management of emphysema has been viewed since then as a potential treatment option for severe emphysema in COPD and since then numerous techniques have been developed, performed, and tested aiming to provide maximum symptom relief and survival benefits and decrease associated morbidity and mortality.

The idea of LVR took origin with Brantigen and colleagues proposing in 1956 the surgical resection of emphysematous portion of lung as a treatment for severe emphysema [19]. However the concept of this modality as an alternative soon faded owing to high perioperative mortality (18%). A special report [20] published in 1996 reviewed 22 articles and case reports dealing with resectional surgery for bullous emphysema since 1950s, which included a total of 476 patients. Early postoperative results showed little improvement in function after surgery for bullae that occupied less than one-third of hemithorax when lung function was normal or minimally impaired. Surgical treatment for severe lung function impairment due to a giant bulla causing compression atelectasis produced the most dramatic improvement. This was mostly associated with type I bullae of paraseptal emphysema, showing modest improvement in FEV1 (forced expiratory volume in 1st second), FEV1/FVC (forced vital capacity) ratio and DLCO (diffusion capacity of lung to carbon-mono-oxide), decreased VC (vital capacity), RV (residual volume) and TLC (total lung capacity), and significant reversal of pulmonary hypertension (PH) and right ventricular hypertrophy (RVH) associated with improvement in hypoxemia and hypercapnia.

In light of these developments and more stringent patient selection, Cooper and colleagues [21] revisited the technique, this time with median sternotomy, and found reduced perioperative mortality (4.8%). They excised 25–30% of volume of each lung using linear stapling device fitted with strips of bovine pericardium to minimize air leak through staple holes [22]. No early or late mortality was observed in this case report of 20 patients. FEV1 showed mean improvement of 82% (0.77 to 1.22 L) and mean increase in PaO_2_ from 66 to 72 mm of Hg on room air.

The work of Cooper et al. [21] revitalized the idea of LVR and encouraged a more dedicated and programmed study (in the form of NETT trial, to be discussed ahead) in the quest of the following hypothesized benefits:

1. improvement in ventilation-perfusion mismatch,
2. a regain of diaphragmatic curvature thereby possibly improving its elasticity,
3. expansion of the compressed normal lung tissue [23],
4. decrease in dynamic hyperinflation and increase in exercise capacity [24],
5. increase in effective intrathoracic volume leading to beneficial effect on left ventricular function,
6. improvement in hypoxemia and hypercapnia with beneficial effect on right ventricular hypertrophy and pulmonary hypertension.

## 3. Techniques in Lung Volume Reduction (LVR)

**Figure 1:**
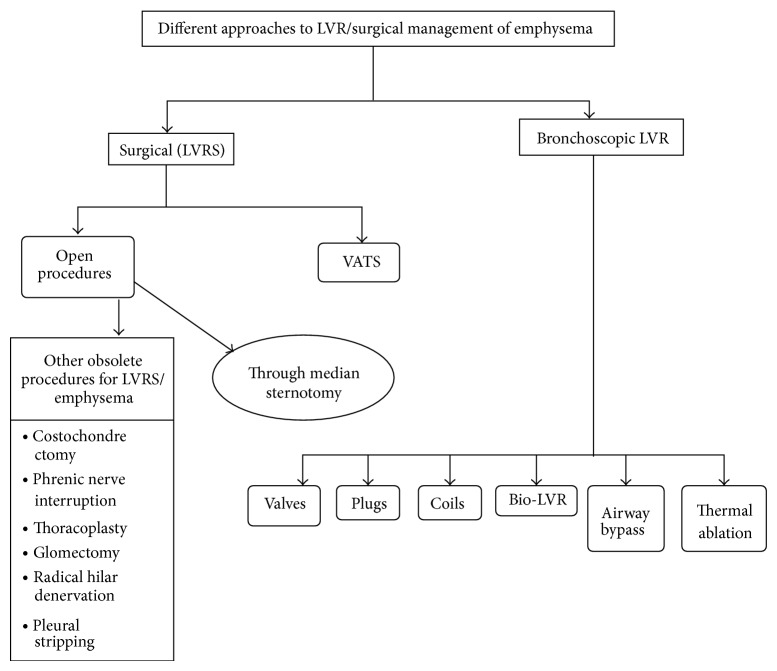
Techniques available for lung volume reduction.

From its advent till now, LVR has come through a long way adapting through various procedures, techniques, and minor and major improvements with the goal to achieve maximum sustainable symptomatic and survival benefits and minimize procedural complications, morbidity, and mortality (see Figure 1).

### 3.1. Surgical LVRS

The National Emphysema Treatment Trial (NETT) [25], supported by National Heart, Lung, and Blood Institute (NHLBI), Centre of Medicare and Medicaid Service (CMS) and Agency for Health care Research and Quality (AHRQ), was the first multicentre clinical trial developed oriented at surgical lung volume resection to achieve two main objectives: (i)* primary objective*, to determine safety and effectiveness of bilateral LVRS in treatment of emphysema, and (ii)* secondary objective*, to develop criteria for identifying patients who are likely to benefit from the procedure.

NETT trial procedures were surgical (nonbronchoscopic) either by bilateral video-assisted thoracoscopic surgery [26], that is, VATS, or median sternotomy. The VATS technique may employ stapling lung resection, Nd-YAG contact laser ablation [27], elastomer sleeves [28] or reinforcement patches [29] using bovine pericardium strips, biologic fibrin glue or blood, and teflon reinforcement patches [28]. It was a landmark study which clarified short-term and long-term risks and benefits of bilateral LVRS to treat severe emphysema (discussed in next section). It was able to predict the following patient characteristics that could predict outcome of surgery [30].

1. Participants mostly with upper-lobe emphysema and low exercise capacity (<25 watts for females and <40 watts for males) were more likely to live longer and more likely to function better after LVRS than after medical treatment. 30% of the surgical group had a 10-watt improvement in exercise capacity compared to none in those treated with medical therapy alone.
2. Participants with mostly upper-lobe emphysema and high exercise capacity had no difference in survival between the LVRS and medical participants, but those in the surgical group were more likely to function better than those who received medical treatment without surgery. Fifteen percent of LVRS participants had more than a 10-watt improvement in exercise compared to three percent of medical participants.
3. Participants with mostly non-upper-lobe emphysema and with low exercise capacity had survival and exercise ability after LVRS similar to that after medical treatment but had less shortness of breath.
4. Participants with mostly non-upper-lobe emphysema and with high exercise capacity had poorer survival after LVRS than after medical treatment, and both LVRS and medical participants had a similar low chance of functioning better.

A thorough retrospective review of case reports, publications, and trials related to lung volume reduction evidently suggests that its role in palliation of symptoms in patients of severe emphysema is significant and may be impressive in a particular group of patients. The liability rests on meticulous patient selection and technical expertise. Below we discuss each procedure with special emphasis on bronchoscopic modalities in light of works done by researchers so far. 

*Lung Volume Reduction Surgery: Analyzing the Physiological Effects, Risks, and Benefits.* The multicentre NETT trial [30] included both median sternotomy and VATS approach depending upon centre and facilities available. It included 1218 patients randomized to either medical treatment or LVRS and confirmed that properly selected patients may experience better functional improvements, exercise capacity, and quality of life than medical treatment,* especially in case of upper-lobe predominant (ULP) disease with poor exercise capacity.* Results broadly concluded as follows.

*Risks*

- Overall mortality over 29-month follow-up: same in LVRS and medical group,
- overall risk of death in first three months: LVRS (7.9%) > medical group (1.3%),
- risk of death in first three months [30] (non-high-risk group): LVRS (5.2%) > medical group (1.5%).

*Benefits*

- More than 10-watt increase in exercise capacity (at 2 years): 15% over all in LVRS (30% in non-high-risk group) >3% in medical group,
- at 2-year follow-up: improved lung function, exercise capacity, 6-minute walk distance (6MWD), quality of life (St. George's Respiratory Questionnaire; SGRQ-score), and dyspnea score with gradual decline to prerandomization level compared to persistent decline of same scores in medical group below prerandomization level.

*Complications*

- 9% intraoperative complication,
- >50% postoperative complications [31] including pneumonia (18.2%), arrhythmia (18.6%), reintubation (21.8%), ICU readmission (11.7%), and tracheostomy (8.2%),
- 7-day (median) air leak in 90% of cases,
- 30-day postoperative hospitalization rate: 28%.

The characterization of patients for fitness for surgery shown in (Table 1) could be made out from NETT research group related studies.

- Patients with upper-lobe predominant emphysema with high baseline exercise capacity showed no survival benefits but an improved exercise capacity from LVRS [30].
- Patients with non-upper-lobe predominant emphysema and high exercise capacity showed no difference in either survival benefits or exercise capacity compared to medical group [30].

### 3.2. Bronchoscopic LVR

The benefits of LVRS evident from the trials and publications infused enthusiasm towards nonsurgical endoscopic approaches to LVR. Bronchoscopic lung volume reduction (BLVR) refers to any of the several bronchoscopic techniques for treating severe emphysema. The following techniques have been in clinical experimentation so far.

#### 3.2.1. Bronchial Valves

**Figure 2:**
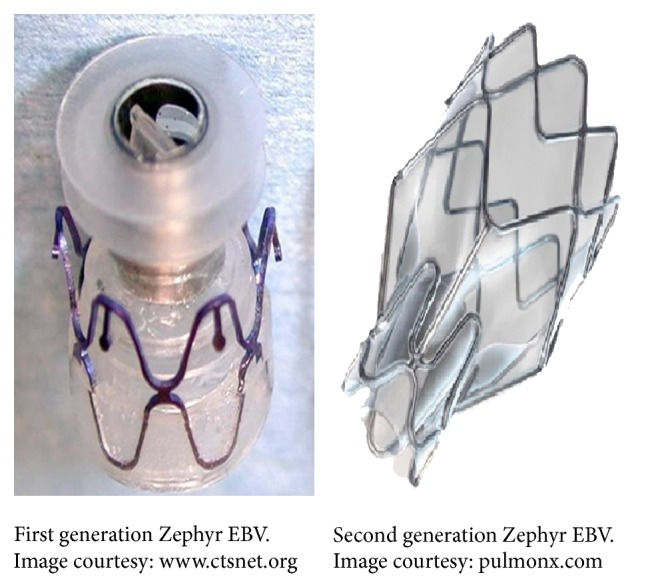
Endobronchial valves. Image courtesy: Pulmonx Inc.

**Figure 3:**
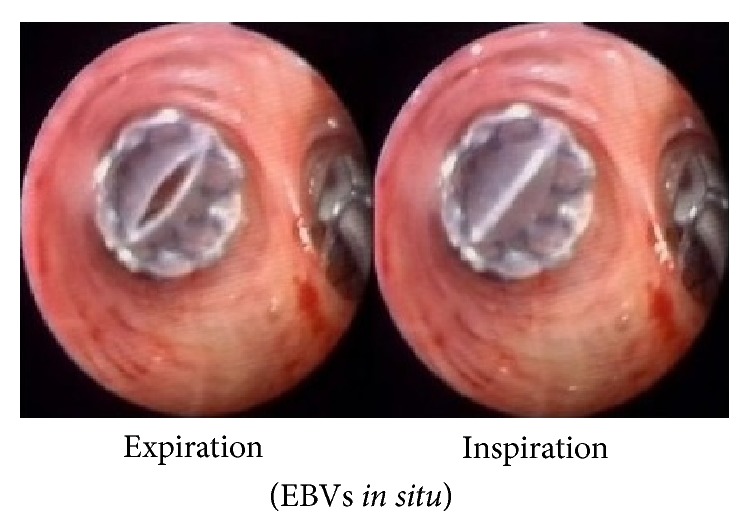
Endobronchial valves placed* in situ*. Image courtesy: Strange et al. [36].

The working principle of endo/intrabronchial valves (EBVs or IBVs) is to allow one-way airflow through the airways they are deployed into; that is, they allow air and secretions to come out while restricting air-entry, thus excluding the nonfunctional emphysematous region from ventilation and reducing dynamic air-trapping. The occluded airway leads to resorption atelectasis of the segments distal to it [34]. There are several types of valves developed till now. Endobronchial valves based on works of Sabanathan et al. [35] are manufactured by Emphasys Medical (California) and Spiration, Inc. (Washington). The Emphasys EBV^R^ (now called Zephyr^R^) is a polymer duck-bill valve mounted inside a stainless steel cylinder attached to a nitinol self-expanding retainer (Figures 2 and 3). It is available in three sizes for different bronchial lumen diameters, 4/5.5 mm, 5/7 mm, and 6.5/8.5 mm with length of 10 mm. The second generation Zephyr EBVs are available in two sizes, “4.0” for bronchial lumen diameter 4–7 mm and “5.5” for 5.5–8 mm lumen diameter, and are supposed to have better expiratory flow rates.

**Figure 4:**
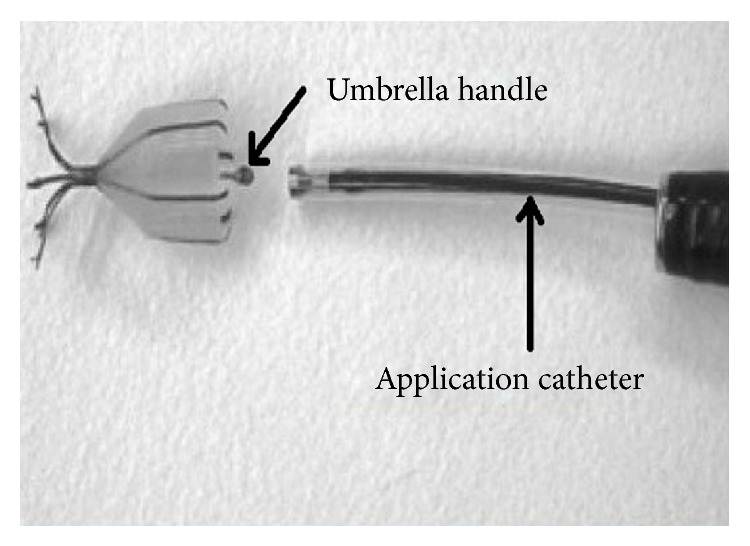
Intrabronchial valve. Image courtesy: Ther. Adv. Resp. Dis. Journal © 2012. London: SAGE.

**Figure 5:**
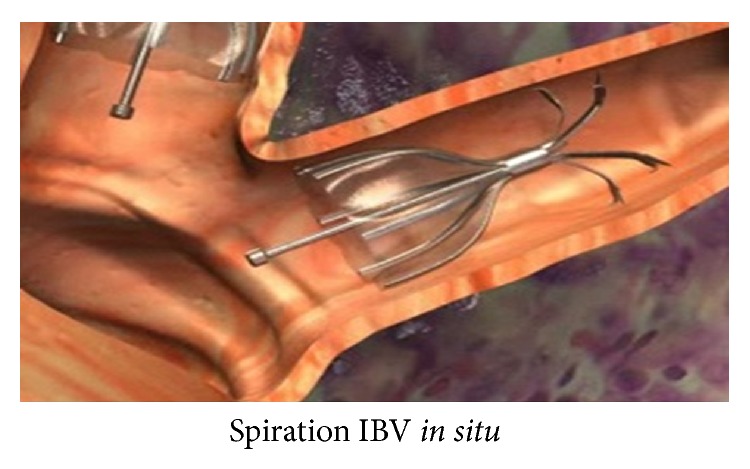
Intrabronchial valve placed* in situ*. Image courtesy: Robert L Berger et al. © 2008: Spiration, Inc.

The Spiration IBV (Figures 4 and 5) has six struts made of nitinol covered by polyurethane membrane in the shape of an umbrella and comes in three sizes with umbrella diameter of 5, 6, or 7 mm. The required size is determined by means of a calibrated balloon catheter and the IBV is inserted at segmental or subsegmental level with the help of an application catheter through the working port of the bronchoscope. It is possible that up to 6 or more valves may be required for the complete blockade of a lobe.

The procedure is usually performed under general anesthesia with patient intubated, on spontaneous assisted ventilation [37]. Though BLVR techniques are now widely being tried and tested, at present the main aim of the research is the identification of suitable patients.

Data on endobronchial valves was limited until the publication of the Endobronchial Valve for Emphysema Palliation (VENT) trial which was a multicentre randomized controlled trial with 220 north-American and 111 European patients enrolled into intervention arm [38–41]. 

*Physiological Effect.* EBVs by occluding airways increased the airway resistance thus diverting air to less emphysematous part of lung and decreasing air-trapping and dynamic hyperinflation [42, 43]. This causes the interlobar shift of ventilation from treated to untreated portion of lung thereby decreasing dead space and increasing ventilation thus reducing previously hypoxia induced vasoconstriction in the healthy areas of lung. This not only improves ventilation-perfusion mismatch but also has beneficial effect on pulmonary hypertension [44, 45]. 

*Benefits* (see [38–41])

- Mean 4.3% improvement in FEV1,
- 6.8% difference in FEV1 in intervention and control arm,
- 5.8% improvement in 6MWD compared to control arm,
- 25.3% improvement in FEV1, 29.9% improvement in 6MWD, and 11-point improvement in SGRQ-score in those patients who had complete fissure and achieved full lobar exclusion,
- symptomatic benefit found in a large subgroup of COPD patients treated with EBVs even in absence of lobar collapse, suggesting role of other physiological mechanism [34, 46, 47],
- patient who had evidence of complete interlobar fissure on preprocedure computed tomography (CT) scan having incremental improvement in FEV1 than those who did not.

*Risks/Drawbacks* (see [38–41])

**Figure 6:**
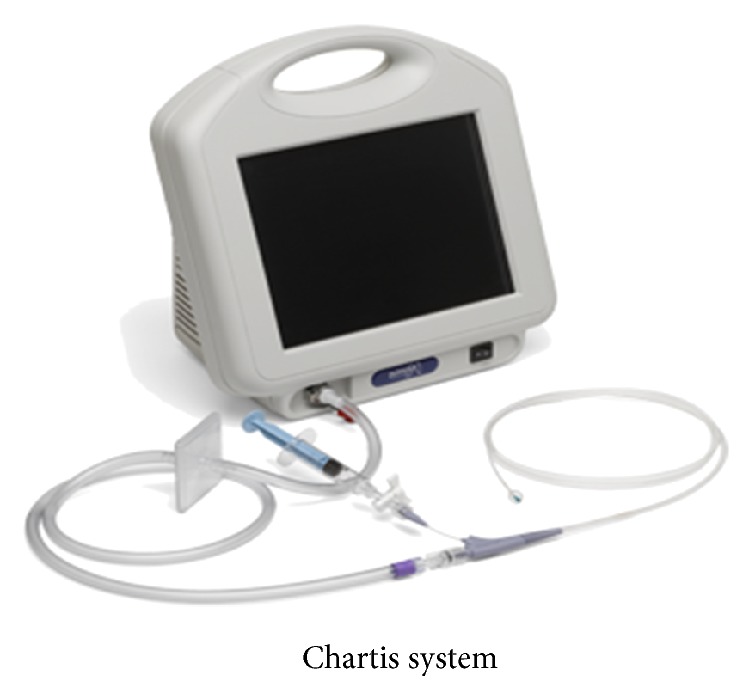
The Chartis Pulmonary Assessment System. Image courtesy: Pulmonx Inc.

- 4.2% composite complication rate at 3 months and 6.1% at 6 months,
- no procedure related mortality documented,
- complications including COPD exacerbation, pneumothorax, hemoptysis, pneumonia, and valve-migration for which valves had to be removed [48],
- >40% of trial centres reported technical error rate of >10%,
- only 22% patients included in VENT trial showing complete interlobar fissure and lobar collapse at 1 year,
- presence of collateral ventilation is a contraindication for this procedure leading to treatment failure. However endobronchial methods (Chartis System, Figure 6) that identify presence of collateral ventilation and thus exclude such patients have also been developed [49].

“High-responders” were considered those who achieved ≥55% CT-scan evident lobar collapse at 6 months.

Sterman et al. [50] and Springmeyer et al. [51] conducted a multicentre pilot study on 98 patients using IBVs (Spiration Inc.) on 98 patients who received bilateral therapy yielding no significant spirometric improvement and 4-point reduction on SGRQ in nearly 50 percent patients with complications like COPD exacerbation and pneumothoraces including one tension pneumothorax related fatality and pneumonia. A multicentre blinded sham-controlled trial [52] of IBVs in* upper-lobe prevalent emphysema without goal of complete lobar occlusion* with 37 patients in intervention arm and 36 without intervention studied the outcomes over 3 months. The results were significant lobar volume shift to treated lobes compared to nontreated lobes with nonsignificant changes in control group. There were ≥4 units of improvement in SGRQ scores in 24% patients of treatment group which were characterized as positive responders while control group yielded no positive responders. Although the procedure was concluded to be safe with no difference in the adverse events reported in the treatment and the control group, it was found to be not effective in majority of patients.

Long-term benefits from BLVR using one way bronchial valves was studied by Kotecha and colleagues [53] in a retrospective cohort study. They studied the outcomes in 23 patients who underwent upper-lobe BLVR from July 2001 to November 2003. With long-term follow-up (≥12 months) seen in 16 of 23 patients with median follow-up of 64 months (range 15 to 90 months), only 6 of these 16 patients remained in follow-up with sustained functional gains at the end of study. Three patients underwent lung transplant in absence of clinical benefits while 4 died during the course of follow-up. Although this demonstrated an acceptable safety profile and possible sustainability of functional gains in selected patients, the study was not controlled against matched medically treated patients to comment on overall survival benefits over the later, a rather small sample size being another concern.

#### 3.2.2. Bronchial Plugs/Occluders/Blockers

Here, devices such as biocompatible sponge, silicon plugs/balloons, and stainless steel stents are used to completely occlude the segmental airway proximal to the emphysematous portion of lung parenchyma. The ventilation distal to it is stopped, causing resorption of the trapped air and sustained collapse of the lung segment [54]. Initial success however failed to yield sustainable long-term results and presented with frequent complications.

#### 3.2.3. Biological Bronchoscopic Lung Volume Reduction (Bio-BLVR)

This is a novel endoscopic technique based on tissue engineering principle which involves instillation of biologically active reagents (chondroitin sulfate, polylysine-fibrin glue and thrombin solution) which leads to replacement of diseased emphysematous tissue by a contracted organized scar thus making it essentially irreversible procedure. Apparent physiological benefits demonstrated from an animal experimental study by Ingenito et al. [55] led to increase in growth of research and commercial interest in this procedure. In their sheep model with papain-induced experimental emphysema, they instilled 10 mL fibrin hydrogel suspension (3% fibrin containing 0.1% chondroitin-6-sulfate and 0.1% poly-L-lysine; Bistech) and 1 mL of thrombin cross-linker (1,000 U thrombin in 1 mL of phosphate-buffered saline containing 5 mm calcium chloride) using a double lumen catheter through a bronchoscope and observed reduced overall lung volume and improved respiratory function safely and consistently. Several procedures followed on human subjects yielding potentially safe results and lesser complications.

**Figure 7:**
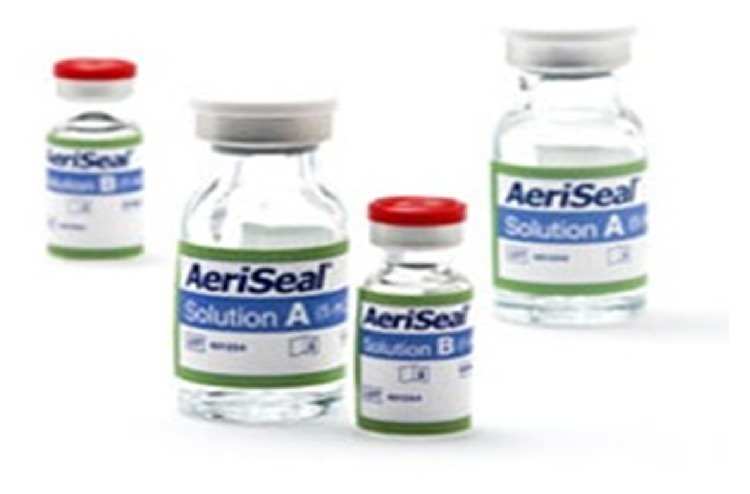
AeriSeal Liquid Foam Sealant. Image courtesy: Aeris Therapeutics.

The Aeris Therapeutics, Woburn, MA, developed its novel tissue sealant, the AeriSeal (Figure 7) which is a liquid foam sealant that collapses hyperinflated lung areas destroyed by emphysema. Pretreatment priming is usually done with primers like porcine trypsin to deactivate surfactants and promote detachment of epithelial cells [56]. The foam of lung sealant AeriSeal is instilled into the peripheral airways and alveoli where it polymerizes and functions as tissue glue, seals the target region, and causes absorption atelectasis [57]. The procedure has been under several trials, the risks and benefits of which are discussed ahead in this paper.

Bronchoscopic injection of autologous blood and fibrinogen into an emphysematous bulla has also effected similar volume reduction [58, 59] but has not been extensively studied except for small pilot projects. The results however are encouraging as they have reported no significant adverse events so far, and the procedure being irreversible may have lasting results and thus warrants a large prospective study.

Bio-LVR procedures using lung sealant as discussed in the previous sections have proven to be most promising by far. Phase-II multicentre trial results published in 2009 by Refaely et al. on 50 patients in bilateral ULP emphysema and another subsequent study [60] concluded the following.

*Risks*

- Serious adverse events in 4 patients that included pneumonia, pulmonary embolism, anesthesia related fall, and aspiration,
- COPD exacerbations in 28% patients, myocardial ischemia and pneumonia in 7%,
- mild postop fever and leukocytosis in nearly 88% of cases in first 24 hours.

*Benefits/Advantages*

- No documented mortality,
- most procedures done on day care basis,
- near similar efficacy and safety profile in homogenous emphysema.

A recent observational study [61] revealed reduction in systemic inflammatory marker C-reactive protein (CRP) following lung sealant induced LVR in emphysema patients, which is now undergoing a large multicentre trial for validation.

#### 3.2.4. Bronchoscopic Thermal Vapour Ablation

**Figure 8:**
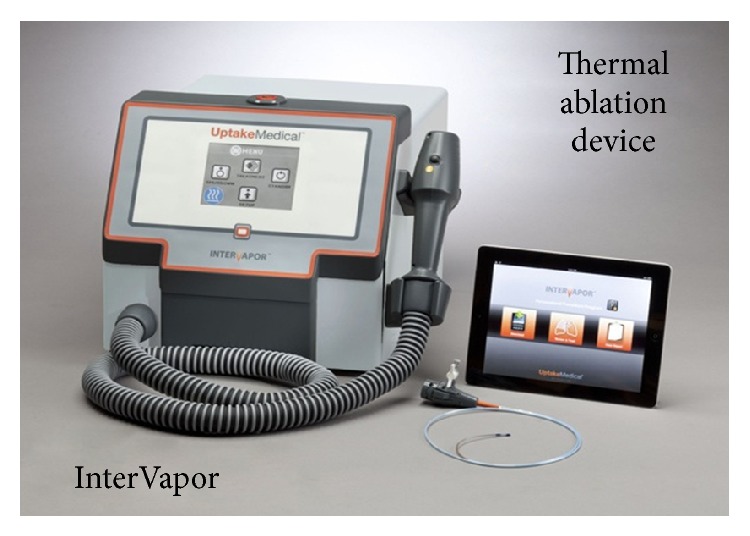
InterVapor Thermal Vapour Ablation Device. Image courtesy: Uptake Medical (Tustin, CA).

Hot vapour/steam application for LVR is still a new modality currently under clinical trials. The preclinical animal studies have provided initial results on safety and efficacy [62, 63]. A specified quantity of steam of quantified heat dose is generated via a steam generator (Figure 8) and delivered through a bronchoscopic catheter. This induces thermal damage and scaring with subsequent absorption atelectasis distal to the bronchus making it inevitably irreversible. Few human studies have been completed with small sample size which has so far demonstrated acceptable safety profile [64, 65]. Though promising, this procedure still has a long way to go before it gets approved.

Not enough data is available to precisely conclude about thermal vapor ablation induced BLVR. A safety and feasibility trial [66] in 11 patients with heterogeneous emphysema recorded no improvement in spirometric measures and a drop in SGRQ-score by 15.3 U. It also recorded incidence of COPD exacerbation in 4 patients and pneumonia in 2 patients. Although it scores an advantage of prosthesis-free procedure and eliminates the concern of collateral ventilation, animal study [30] suggests that there may be long-term squeals like pleural adhesion and heat induced damage to adjacent structures like phrenic nerve, pericardium, and so forth. A study by Herth et al. [67] to study one-year postintervention outcomes provides some insight into the long-term outcomes. This multicentre single arm treatment trial included 44 patients with upper-lobe emphysema of mean ± standard deviation age 63 ± 5.6 years and FEV_1_  0.86 ± 0.25 mL. Treatment outcome characterization at 12 months yielded markedly significant improvement in FEV_1_, St. George's Respiratory Questionnaire and Modified Medical Research Council Scores, lobar volume shift (measured by high resolution computed tomography) RV, and 6-minute walk distance. The improvements were significantly larger from baseline but lower numerically from those at 6-month follow-up. The improvements were greater in GOLD stage IV patients and emphysema with higher heterogeneity. The results clearly show sustained functional gains but survival benefits as compared to medical treatment groups have not been assessed. Further prospective trials are warranted to study the same.

#### 3.2.5. Endobronchial Coils

**Figure 9:**
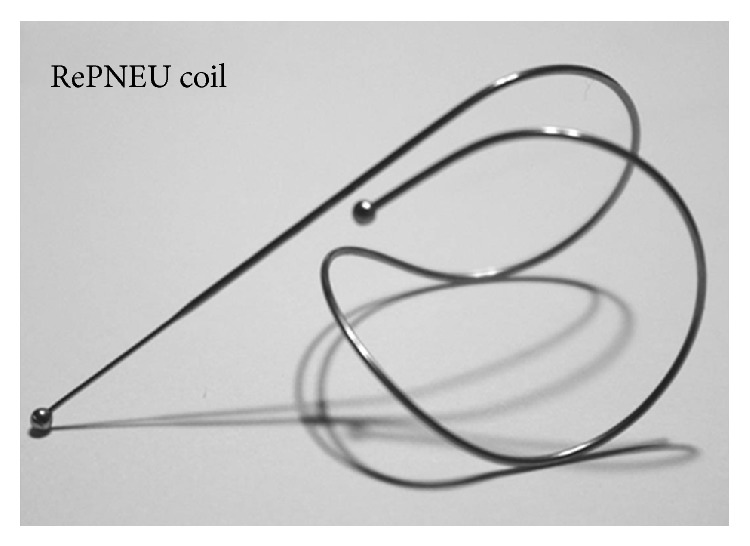
RePneu Coil. Image courtesy: PneuRx Incorporated, USA.

The novel RePneu lung volume reduction coil (LVRC) is a new medical device developed by PneuRx Incorporated, Mountainview, CA, USA (Figure 9). It is a self-activating metal (nitinol) coil which is placed (in straightened form) bronchoscopically into the most diseased regions in severe emphysema using a proprietary delivery system, but when released in the lung it springs back into the predetermined coiled shape, gathering the lung tissue around it. The device was CE (Conformité Européenne) marked in Europe in October 2010 but is not currently available within the NHS (National Health Service, United Kingdom) outside of research.

To date there are no published trials which assess how effective treatment using RePneu LVRCs is, compared to other LVR treatments for emphysema. Two noncomparative studies have been completed [68, 69] and published. A study to demonstrate safety and performance of the LVRCs in patients with emphysema (RESET study) has recently completed its phase-I trial and reported no difference in serious adverse events between LVRC treatment and medical care group [70, 71]. Another ongoing randomized controlled trial to study safety and efficacy of LVRC system is in phase-III [72].

This new novel technique as described above is still under clinical trial and has yielded results with acceptable safety and efficacy margins. A recent prospective cohort pilot study published in November 2011 [69], including 16 patients, showed after 6 months of treatment significant improvement in the following:

- SGRQ (−14.9 ± 12.1, with 11 patients improving >4 points),
- FEV1 (+14.9% ± 17.0%), FVC (+13.4% ± 12.9%), and RV (−11.4% ± 9.0%),
- 6MWD (+84.4 m ± 73.4 m).

The procedure is still in its infancy and awaits further research.

#### 3.2.6. Bronchial Fenestration and Airway Bypass

**Figure 10:**
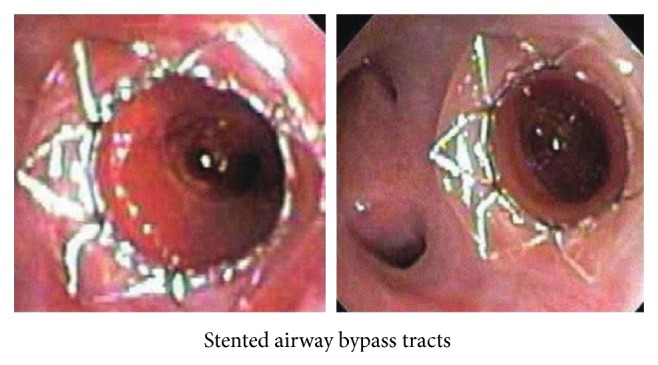
Stented airway bypass tracts. Image courtesy: Ther Adv Resp Dis 2012: London: SAGE.

In this technique, a collateral bypass tract is created between the distal emphysematous portion of lung and bronchus. Pretreatment assessment of segmental bronchi is done using a Doppler probe to find an area free from blood vessel. Then using noncollapsible, low-resistance, paclitaxel-eluting bronchial stents a new conducting expiratory airway is created (Figure 10). This overcomes the problem of collateral ventilation which is a contraindication for other BLVR techniques. This technique though developed targeting patients with homogenous emphysema has not been so successful as hypothesized because it is a complex technique that is not significantly without risks and has shown no long-term improvements when studied [73, 74].

A very innovative and theoretically logical approach, bronchial fenestration airway bypass technique, was hypothesized to be useful in homogenous emphysema with significant collateral ventilation. However, a recent multicentre open label study on 35 patients [75] and a double-blind study of 208 patients [76], the EASE (Exhale Airway Stents for Emphysema) trial, yielded at 6 months: limited efficacy, no significant spirometric change, no improvement in SGRQ-score, and stent patency being retained in only 24% to 69% cases. Besides, it has limitations including risk of bleeding, specialized training and equipment, and tendency of the radiofrequency ablation pathways to close thereby limiting long-term effectiveness of this procedure. The benefits after all could not promise long-term sustainability.

## 4. Conclusion: The Journey Travelled and Roads Ahead

It is a matter of joy that we have come a long way in quite a short span of time since the advent of BLVR. We tried, we failed, we succeeded, and, above all, we learned from our and others mistakes. There is no doubt the BLVR has added a new dimension to the treatment of emphysema and it is going to be a long player. Now we are in a position better than ever to define the indications and contraindications for selecting the group of patients which can benefit most from the technique [77]. We can consolidate them in the following best way which can definitely benefit the intervention pulmonologists.

- * Pretreatment Assessment.*
  - Do a high resolution CT scan to morphologically classify emphysema. The most seriously affected region can be better visualized using additional computer programs (e.g., YACTA). A pulmonary perfusion scan can be used to record distribution of ventilation and perfusion.
  - A complete pulmonary function test must be performed to determine all lung function parameters.
  - St. George's quality of life survey and exercise capacity testing (using bicycle method) must be performed to monitor the treatment outcome.
  - Degree of collateral ventilation can be determined using Chartis System.
- * Patient Selection.*
  - Heterogeneous and ULP emphysema bear better prognosis especially if with low exercise capacity.
  - Best responders are those with higher degree of air-trapping, residual volume (RV) >225% of predicted, TLC >150% of predicted, FEV1 20–45%, and diffusion capacity for carbon monoxide (DLCO) 20–59%.
  - FEV1 <20% and DLCO <20% of predicted are a strict contraindication for BLVR.
- * Procedure Selection.*
  - In presence of complete fissural integrity (complete lobar exclusion) and no collateral ventilation, endobronchial valves may be preferred owing to their reversible nature, so that they can be removed if the procedure does not work or the condition of patient worsens.
  - In presence of collateral ventilation in heterogeneous emphysema or when emphysema is homogeneous, lung sealants or coil implants may be better suited as the work at parenchyma level.

In the end, treating interventionist's discretion is expertise and experience holds the final call. There also remains a scope to look at the existing techniques with new perspectives.

Despite a large number of trials and literatures available there have not been studies focusing on comparing the survival benefits from BLVR over medical treatment group in long-term follow-up. The sample size for this purpose needs to be very large in order to yield a statistically reliable result. This could be possible if researchers from multiple centers collaborate to work up a single uniform study protocol and results/data collected thereafter could be pooled in together which could serve as an international database for bronchoscopic lung volume reduction. This database would also help to compare the benefits and risks of each individual type of bronchoscopic procedures.

Beyond what we have learned so far, we must strive for further improvement in the present techniques and criteria and developing newer and cost-effective procedures as well and not to forget that all the present modalities are too expensive. Meanwhile, BLVR has a lot of promises to keep.

## Figures and Tables

**Table 1:** Patient characterization for LVRS (adapted with modification [32]).

Indication (non-high-risk, better outcome chance)	Contraindication (high-risk)
(1) Age <75 years (2) Marked dyspnea; MRC score >3 (3) Severe emphysema: hyperinflation: TLC >125% of predicted, RV/TLC >0.65, and FEV1 <35% of predicted (4) Upper-lobe predominant emphysema (ULP) with low exercise capacity	(1) Severe parenchymal loss with DLCO <20% of predicted (2) FEV1 <20% (3) Pulmonary hypertension; mean pulmonary artery wedge pressure >35 (4) Coronary artery disease. (5) Homogenously [33] distributed emphysema
